# Lung, CNS and musculo-skeletal targeted therapy-induced toxicity: imaging features

**DOI:** 10.1186/1470-7330-14-S1-O38

**Published:** 2014-10-09

**Authors:** Nina Tunariu

**Affiliations:** 1 Department of Radiology, Royal Marsden NHS Foundation Trust, Sutton, SM2 5PT, London, UK

## 

By focusing on molecular and cellular changes that are up regulated in cancer, targeted agents may have less severe side effects compared with conventional chemotherapy. However, many of these signaling pathways also exist in healthy cells and targeted therapies can therefore result in specific complications [1-3].

The spectrum of toxicity encountered with the targeted agents includes fatigue, skin rash and mucositis, eye, cardiac and muscle toxicity, drug-induced interstitial lung disease (DILD), posterior reversible encephalopathy syndrome (PRES) and a wide spectrum of gastro-intestinal complications. Nephrotoxic effects such as proteinuria, nephrotic syndrome or hypertension are also seen with agents such as VEGFR or mTOR inhibitors.

The DILD imaging appearances encompass a range of patterns including nonspecific ground-glass opacity with and without interlobular septal thickening, multifocal consolidations with or without traction bronchiectasis, flitting subcentimetre nodules with or without ground glass halo, bronchiolitis and pulmonary fibrosis or pleural effusions [4]. Acute respiratory distress syndrome (ARDS) has also been described. The EGFR inhibitors related DILD is an important challenge for their use in lung carcinoma especially in patients with preexisting pulmonary fibrosis. Up to one-sixth of patients taking mammalian target of rapamycin (mTOR) inhibitors get a reversible interstitial pneumonitis [5].

Posterior reversible encephalopathy syndrome (PRES) is an uncommon complication (incidence < 0.1%) of VEGF/VEGFR pathway therapy. Most patients recover fully with discontinuation of the causative agent and supportive management of hypertension and seizures [6]. Intra-cerebral bleeding has also been described with anti-VEGF agents, especially in tumours with a strong hemorrhagic tendency, such as renal-cell carcinoma. On imaging, there is usually symmetrical vasogenic oedema in the occipital and parietal regions. However, PRES can be found in a non-posterior distribution, occurring at watershed areas of vascular territories, including the frontal, inferior temporal, cerebellar and brainstem areas.

Increases of creatine kinase (CK) are amongst the most common grade 3-4 toxicity treatment-related adverse events of the Mitogen-activated protein kinase (MEK) inhibitors, although most patients are asymptomatic. Symptoms of CK increase include muscle weakness and myalgia. The true significance especially of asymptomatic CK increases is still unknown. An association between CK elevation and skin rash of novel targeted agents in phase I trials, possibly due to increased CK-BB (brain and skin) expression of keratinocytes was reported by Moreno Garcia et al [7].

With the continuous increase in the use of anti-cancer targeted therapies, it is important for the radiologist to be familiar with the clinical and treatment history and associated complications that may occur, in order to accurately identify drug-related complications and differentiate them from disease progression.
